# Geographic Variation in Qualified Health Plan Coverage and Prior Authorization Requirements for HIV Preexposure Prophylaxis

**DOI:** 10.1001/jamanetworkopen.2023.42781

**Published:** 2023-11-10

**Authors:** Kathleen A. McManus, Benjamin Fuller, Amy Killelea, Andrew Strumpf, Samuel D. Powers, Elizabeth T. Rogawski McQuade

**Affiliations:** 1 Division of Infectious Diseases and International Health, University of Virginia, Charlottesville; 2 Department of Medicine, University of Virginia, Charlottesville; 3 Killelea Consulting, Arlington, Virginia; 4 Public Health Sciences, University of Virginia, Charlottesville; 5 Rollins School of Public Health, Emory University, Atlanta, Georgia

## Abstract

**Importance:**

HIV preexposure prophylaxis (PrEP) is a key component of the Ending the HIV Epidemic (EHE) Initiative to curb new HIV diagnoses. In October 2019, emtricitabine/tenofovir alafenamide was added as an approved formulation for PrEP in addition to emtricitabine/tenofovir disoproxil fumarate; despite availability of another formulation with a similar prevention indication, variations in coverage may limit access.

**Objective:**

To assess qualified health plan (QHP) coverage, prior authorization (PA) requirements, and specialty tiering for emtricitabine/tenofovir disoproxil fumarate and emtricitabine/tenofovir alafenamide following emtricitabine/tenofovir alafenamide approval as a PrEP treatment.

**Design, Setting, and Participants:**

This cross-sectional study analyzed QHPs in the US that were compliant with the Patient Protection and Affordable Care Act from 2018 to 2020. QHPs were categorized by region and EHE priority jurisdictions. Data analysis occurred from March 2022 to March 2023.

**Exposures:**

Enrollment in a qualified health plan certified by the Patient Protection and Affordable Care Act.

**Main Outcome and Measures:**

Annual variation in QHP coverage and PA requirement for emtricitabine/tenofovir disoproxil fumarate and/or emtricitabine/tenofovir alafenamide. Descriptive statistics were reported for all outcomes. A secondary outcome was whether the PrEP formulation was determined by the QHP to be placed on a specialty drug tier.

**Results:**

A total of 58 087 QHPs (19 533 for 2018; 17 007 for 2019; and 21 547 for 2020) were analyzed. QHPs covered emtricitabine/tenofovir disoproxil fumarate (19 165 QHPs [98.1%] in 2018; 16 970 QHPs [99.8%] in 2019; 20 045 QHPs [94.8%] in 2020) at a higher rate than emtricitabine/tenofovir alafenamide (17 391 QHPs [91.9%] in 2018; 15 757 QHPs [92.7%] in 2019; 18 836 QHPs [87.4%] in 2020). QHPs in the South required exclusive PA (ie, PA for 1 of the formulations even if the QHP covered both) for emtricitabine/tenofovir disoproxil fumarate and emtricitabine/tenofovir alafenamide at the highest rates in all 3 years. In the South, the rate of PA for emtricitabine/tenofovir disoproxil fumarate increased from 806 of 8023 QHPs (10.0%) in 2018 to 3466 of 7401 QHPs (46.8%) in 2020. QHPs with exclusive PA requirement for emtricitabine/tenofovir disoproxil fumarate were higher in EHE jurisdictions than non–EHE jurisdictions (difference: 2018, 0.9 percentage points; 2019, 3.5 percentage points; 2020, 29.1 percentage points). QHPs were more likely to place emtricitabine/tenofovir disoproxil fumarate on a specialty tier compared with emtricitabine/tenofovir alafenamide (difference: 2018, 1.8 percentage points; 2019, 3.7 percentage points; 2020, 4.1 percentage points).

**Conclusions and Relevance:**

In this cross-sectional study, despite similar indications for biomedical prevention, QHPs were more likely to cover emtricitabine/tenofovir disoproxil fumarate than emtricitabine/tenofovir alafenamide, and QHPs were also more likely to subject emtricitabine/tenofovir disoproxil fumarate to PA or place it on a specialty tier despite the broader clinical indication. QHP PA requirements of emtricitabine/tenofovir disoproxil fumarate following emtricitabine/tenofovir alafenamide approval does not reflect clinical guidelines. The requirements could reflect differences in clinical indication, manufacturer discounts, or anticipation of a changing regulations and emerging generics. High rates of exclusive PA for emtricitabine/tenofovir disoproxil fumarate in areas where rates of HIV diagnoses are highest and PrEP is most needed (eg, the South and EHE priority jurisdictions) is concerning; policy solutions to address the growing PrEP health equity crisis could include regulator actions and a national PrEP program.

## Key Points

**Question:**

How do qualified health plan (QHP) coverage and prior authorization (PA) requirements for HIV preexposure prophylaxis (PrEP) differ across regions of the US?

**Findings:**

In this cross-sectional study of 58 087 QHPs, QHPs in the South and Midwest had the highest rate of requiring PA for emtricitabine/tenofovir disoproxil fumarate but not emtricitabine/tenofovir alafenamide among all regions, and the emtricitabine/tenofovir disoproxil fumarate PA requirement rate nearly doubled from 2019 to 2020. Despite broader clinical indications, the emtricitabine/tenofovir disoproxil fumarate PA requirement rate was higher than that for emtricitabine/tenofovir alafenamide, most notably in Ending the HIV Epidemic Initiative priority jurisdictions.

**Meaning:**

These findings suggest that high rates of PA requirement may directly impede PrEP access and uptake in geographic areas with high rates of HIV diagnoses.

## Teaser

This cross-sectional study analyzes coverage and prior authorization requirements for HIV preexposure prophylaxis among qualified health plans from 2018 to 2020.

## Introduction

Biomedical HIV prevention with HIV preexposure prophylaxis (PrEP) has been approved by the US Food and Drug Administration (FDA) since 2012, and if widely used, has the possibility to help end the HIV epidemic in the US.^1,2,3^ PrEP efficacy at reducing HIV acquisition exceeds 90% for sexual encounters and exceeds 70% for injection drug use.^4^ However, PrEP use is very low compared with PrEP need, particularly in the South, where adverse social determinants of health can exacerbate health inequities.^5,6,7^ Frequently cited barriers in the South for low PrEP use include lack of clinicians with PrEP knowledge, lack of health insurance, stigma, and underestimation of personal HIV risk.^8,9,10,11,12^ Perceived and actual high costs to the patient are also barriers to PrEP use.^13,14,15^ Costs include deductibles, copayments, and coinsurance for medications and the ancillary services necessary for PrEP, including laboratory services and clinic visits. Improved biomedical prevention through access to PrEP is 1 of the essential components of the Ending the HIV Epidemic (EHE) Initiative.^3^

From 2012 to 2019, there was only 1 medication approved by the FDA for PrEP: combined emtricitabine/tenofovir disoproxil fumarate, manufactured by Gilead Sciences under the brand name Truvada. In October 2019, the FDA approved a new formulation of PrEP, combined emtricitabine/tenofovir alafenamide, which is manufactured by Gilead Sciences under the brand name Descovy.^16^ The FDA approval for emtricitabine/tenofovir alafenamide excluded individuals who have receptive vaginal sex due to insufficient data about safety and efficacy among cisgender women and transgender men who have receptive vaginal intercourse.^17^

Clinical data has not shown emtricitabine/tenofovir alafenamide to be superior to tenofovir disoproxil fumarate-based regimens. Data has shown emtricitabine/tenofovir alafenamide to be associated with weight gain and lipid increases compared with tenofovir disoproxil fumarate–based regimens, and emtricitabine/tenofovir alafenamide may be preferable for those with preexisting kidney or bone disease and those who experience kidney or bone adverse events while using emtricitabine/tenofovir disoproxil fumarate.^18^ As of 2020, both brand-name emtricitabine/tenofovir disoproxil fumarate and emtricitabine/tenofovir alafenamide wholesale acquisition costs or list price were identical at $1823 per month.^19^

Although not having insurance is a barrier to obtaining PrEP, there is evidence of insurance companies using benefit designs to discourage people from accessing expensive medications^20^ to minimize company costs. These benefit designs include prior authorization (PA), in which a clinician must get plan approval before prescribing a certain medication, and specialty tiering (ie, when a medication is placed on a high cost-sharing tier and may have to be mailed by a specialty pharmacy). Qualified health plans (QHPs) are health insurance plans that are certified by the Patient Protection and Affordable Care Act (ACA) health insurance marketplaces and meet federal criteria.^21^ With more than 11 million Americans relying on QHPs for health care access, any disparities in the benefit design of QHPs for PrEP access could have far-reaching effects.^22^ During the time when there was only 1 formulation of PrEP available, our research group found regional disparities in PA requirements for PrEP, with plans in the South being almost 16 times as likely as plans in the Northeast to require PA.^23^

The PrEP landscape continues to evolve. In late 2020, the first manufacturer began marketing generic emtricitabine/tenofovir disoproxil fumarate under a 6-month exclusivity deal.^24^ Multiple manufacturers began marketing generic emtricitabine/tenofovir disoproxil fumarate in late spring 2021,^25^ and long-acting, injectable cabotegravir was approved in December 2021.^26^ For the 2 available PrEP formulations from 2018 to 2020, we sought to characterize insurance coverage, PA, and specialty tiering by geography, including regions and EHE priority jurisdictions.

## Methods

### Data

This cross-sectional study was approved by the University of Virginia institutional review board with a waiver of informed consent because it constituted nonhuman participants research in accordance with 45 CFR 46. This study is reported following the Strengthening the Reporting of Observational Studies in Epidemiology (STROBE) reporting guideline. We used the Robert Wood Johnson Foundation 2018 to 2020 Individual Market Health Insurance Exchange Compare data set linked with Ideon plan-level formulary data sets. Data were obtained from Ideon through a data use agreement.

A unique QHP was defined as an ACA–compliant individual plan offered on the ACA marketplace during at least 1 of the calendar years (2018, 2019, and 2020) in a specific rating area; geographic boundaries were defined by the US Department of Health and Humans Services (HHS). We excluded cost-sharing reduction (ie, income-based variations of an on-market plan) and child only variants of QHPs. QHPs offered in the same state with identical cost-sharing benefits but in different rating areas were considered unique plans. Using their corresponding rating area, QHPs were categorized by region using US Census Bureau definitions (Northeast, Midwest, West, and South).^27^ Using the HHS list of EHE Phase 1 priority jurisdictions (48 counties; San Juan, Puerto Rico; Washington, District of Columbia; and 7 states) where HIV diagnoses occur most frequently or with substantial rural burden, we categorized QHPs as being in a rating area located in an EHE priority jurisdiction or not.^28^

### Outcomes

Annual variation among QHPs in coverage and PA requirement for emtricitabine/tenofovir disoproxil fumarate and emtricitabine/tenofovir alafenamide were our primary outcomes. A secondary outcome was whether the PrEP formulation was determined by the QHP to be placed on a specialty drug tier.

To evaluate plan characteristics, we analyzed each QHP on the basis of the variation in coverage, PA requirements, and specialty tiering of emtricitabine/tenofovir disoproxil fumarate and emtricitabine/tenofovir alafenamide. First, we quantified the QHPs that covered emtricitabine/tenofovir disoproxil fumarate and those that covered emtricitabine/tenofovir alafenamide. We then categorized each QHP on the basis of discrete levels of coverage: both formulations covered, only emtricitabine/tenofovir disoproxil fumarate (ie, exclusive coverage of emtricitabine/tenofovir disoproxil fumarate and not emtricitabine/tenofovir alafenamide), only emtricitabine/tenofovir alafenamide (ie, exclusive coverage of emtricitabine/tenofovir alafenamide and not emtricitabine/tenofovir disoproxil fumarate), or neither formulation. Once categorized and reported at the national level, we stratified the QHPs on the basis of geographic areas (regional and EHE vs non-EHE). To evaluate PA patterns, we analyzed QHPs covering both formulations, determined whether they required PA, categorized them using the same levels for coverage (both formulations, only emtricitabine/tenofovir disoproxil fumarate, only emtricitabine/tenofovir alafenamide, and neither), and stratified them on the basis of geographic area (national, regional, and EHE vs non–EHE). To evaluate specialty tiering patterns, we analyzed QHPs covering emtricitabine/tenofovir disoproxil fumarate or emtricitabine/tenofovir alafenamide, determined whether the covered formulation was placed on a specialty tier, and stratified them on the basis of geographic area (national, regional, and EHE vs non–EHE).

### Statistical Analysis

Descriptive statistics were reported for all outcomes. We did not estimate the uncertainty around prevalence estimates because the data set included all QHPs. Analyses were performed using R statistical software version 4.1.3 (R Project for Statistical Computing) and RStudio version 1.4.1743-4 (Posit). Data analysis occurred from March 2022 to March 2023.

## Results

### Overall Coverage and PA

A total of 58 087 QHPs were analyzed, including 19 533 for 2018, 17 007 for 2019, and 21 547 for 2020. eTable 1 in Supplement 1 reports national coverage and PA requirements. Nationally, QHPs covered emtricitabine/tenofovir disoproxil fumarate at a higher rate than emtricitabine/tenofovir alafenamide from 2018 to 2020 (19 165 QHPs [98.1%] vs 17 931 QHPs [91.8%] in 2018; 16 970 QHPs [99.8%] vs 15 757 QHPs [92.7%] in 2019; and 20 425 QHPs [94.8%] vs 18 836 QHPs [87.4%] in 2020). Among QHPs that covered both formulations, QHPs required PA for emtricitabine/tenofovir disoproxil fumarate at a higher rate than emtricitabine/tenofovir alafenamide fumarate (4154 of 19 165 QHPs [21.7%] vs 2600 of 17 931 QHPs [14.5%] in 2018; 3232 of 16 970 QHPs [19.0%] vs 1711 of 15 757 QHPs [10.9%] in 2019; and 7670 of 20 425 QHPS [37.6%] vs 1657 of 18 836 QHPS [8.8%] in 2020). Table 1 demonstrates national coverage and PA of both formulations, only emtricitabine/tenofovir disoproxil fumarate, only emtricitabine/tenofovir alafenamide, and neither. Prevalence of QHPs that required PA on both PrEP formulations declined over the 3 years (2573 QHPs in 2018 [14.4%]; 1711 QHPs in 2019 [10.9%]; and 1647 QHPs in 2020 [8.8%]).

**Table 1.** National Access to F/TDF and F/TAF Among Qualified Health Plans, 2018-2020

Accessibility	Qualified health plans, No. (%) (N = 58 087)		
	2018 (n = 19 533)	2019 (n = 17 007)	2020 (n = 21 547)
**Coverage**			
Both F/TDF and F/TAF	17 814 (91.2)	15 757 (92.7)	18 692 (86.7)
Only F/TDF	1351 (6.9)	1213 (7.1)	1733 (8.0)
Only F/TAF	117 (0.6)	0	144 (0.7)
Neither	251 (1.3)	37 (0.2)	978 (4.5)
**Prior authorization requirement** ^a^			
Both F/TDF and F/TAF	2573 (14.4)	1711 (10.9)	1647 (8.8)
Only F/TDF	1581 (8.9)	1521 (9.7)	6023 (32.2)
Only F/TAF	117 (0.6)	0	10 (0.1)
Neither	13 633 (76.5)	12 525 (79.5)	11 012 (58.9)

Abbreviations: F/TAF, emtricitabine/tenofovir alafenamide; F/TDF, emtricitabine/tenofovir disoproxil fumarate.

^a^ Prior authorization assessed for plans that covered both F/TDF and F/TAF.

### Coverage and PA by Region

eTable 2 in Supplement 1 demonstrates coverage and PA requirements stratified by region. Table 2 and Table 3 demonstrate regional coverage and PA for both formulations, only emtricitabine/tenofovir disoproxil fumarate, only emtricitabine/tenofovir alafenamide, or neither. eTables 3 to 6 in Supplement 1 report coverage and PA requirements by state.

**Table 2.** Regional Coverage of F/TDF and F/TAF Among Qualified Health Plans, 2018-2020

Coverage by year	Qualified Health Plans, No./Total No. (%)			
	Northeast	Midwest	South	West
Both F/TDF and F/TAF				
2018	2574/3183 (80.9)	4332/4650 (93.2)	8023/8470 (94.7)	2885/3230 (89.3)
2019	2851/3106 (91.8)	3824/4210 (90.8)	5991/6341 (94.5)	3091/3350 (92.3)
2020	2872/3486 (82.4)	5147/5535 (93.0)	7401/8505 (87.0)	3272/4021 (81.4)
Only F/TDF				
2018	593/3183 (18.6)	261/4650 (5.6)	338/8470 (4.0)	159/3230 (4.9)
2019	218/3106 (7.0)	386/4210 (9.2)	350/6341 (5.5)	259/3350 (7.7)
2020	468/3486 (13.4)	217/5535 (3.9)	602/8505 (7.1)	446/4021 (11.1)
Only F/TAF				
2018	0	0	0	117/3230 (3.6)
2019	0	0	0	0
2020	0	0	0	144/4021 (3.6)
Neither				
2018	16/3183 (0.5)	57/4650 (1.2)	109/8470 (1.3)	69/3230 (2.1)
2019	37/3106 (1.2)	0	0	0
2020	146/3486 (4.2)	171/5535 (3.1)	502/8505 (5.9)	159/4021 (4.0)

Abbreviations: F/TAF, emtricitabine/tenofovir alafenamide; F/TDF, emtricitabine/tenofovir disoproxil fumarate.

**Table 3.** Regional Prior Authorization Requirements of F/TDF and F/TAF Among Qualified Health Plans, 2018-2020^a^

Prior authorization by year	Qualified Health Plans, No./Total No. (%)			
	Northeast	Midwest	South	West
Both F/TDF and F/TAF				
2018	0	236/4332 (5.4)	2307/8023 (28.8)	30/3230 (1.0)
2019	64/2851 (2.2)	81/3824 (2.1)	1534/5991 (25.6)	32/3350 (1.0)
2020	0	79/5147 (1.5)	1538/7401 (20.8)	30/4021 (1.0)
Only F/TDF				
2018	20/2574 (0.8)	535/4332 (12.3)	806/8023 (10.0)	220/3230 (7.6)
2019	8/2851 (0.3)	481/3824 (12.6)	850/5991 (14.2)	182/3350 (5.9)
2020	232/2872 (8.1)	2036/5147 (39.6)	3466/7401 (46.8)	289/4021 (8.8)
Only F/TAF				
2018	0	27/4332 (0.6)	0	0
2019	0	0	0	0
2020	0	5/5147 (0.1)	0	5/4021 (0.2)
Neither				
2018	2554/2574 (99.2)	3534/4332 (81.6)	4910/8023 (61.2)	2635/3230 (91.3)
2019	2779/2851 (97.5)	3262/3824 (85.3)	3607/5991 (60.2)	2877/3350 (93.1)
2020	2640/2872 (91.9)	3027/5147 (58.8)	2397/7401 (32.4)	2948/4021 (90.1)

Abbreviations: F/TAF, emtricitabine/tenofovir alafenamide; F/TDF, emtricitabine/tenofovir disoproxil fumarate.

^a^ Prior authorization assessed for plans that covered both F/TDF and F/TAF.

#### Northeastern Region

Both formulations were covered at different rates from 2018 to 2020. Coverage of only emtricitabine/tenofovir disoproxil fumarate also varied by year. No QHPs exclusively covered emtricitabine/tenofovir alafenamide. PA for both formulations was rare. QHPs with PA on only emtricitabine/tenofovir disoproxil fumarate increased over time. No QHPs exclusively required PA on emtricitabine/tenofovir alafenamide. Most QHPs did not require PA on either formulation from 2018 to 2020.

#### Western Region

Coverage of both formulations was high from 2018 to 2020. Exclusive coverage of emtricitabine/tenofovir disoproxil fumarate increased, whereas exclusive coverage of emtricitabine/tenofovir alafenamide was lower. QHPs required PA on both formulations at consistently low rates. Exclusive PA for emtricitabine/tenofovir disoproxil fumarate was high compared with exclusive PA for emtricitabine/tenofovir alafenamide, which was rare (difference: 2018, 7.6 percentage points; 2019, 5.9 percentage points; 2020, 8.6 percentage points). PA on neither formulation was common.

#### Midwestern Region

From 2018 to 2020, coverage of both PrEP formulations was common among QHPs. Exclusive coverage of emtricitabine/tenofovir disoproxil fumarate varied, whereas there were no QHPs that only covered emtricitabine/tenofovir alafenamide. Prevalence of QHPs with PA requirements on both formulations declined. Exclusive PA for emtricitabine/tenofovir disoproxil fumarate increased, whereas exclusive PA for emtricitabine/tenofovir alafenamide remained low. PA requirements on neither formulation were common in 2018 and 2019 but declined by 2020.

#### Southern Region

In the South, from 2018 to 2020, coverage of both PrEP formulations was high. Exclusive coverage of emtricitabine/tenofovir disoproxil fumarate increased from 806 of 8023 QHPs (10.0%) in 2018 to 3466 of 7401 QHPs (46.8%) in 2020. There were no QHPs that covered only emtricitabine/tenofovir alafenamide. In contrast with other regions, PA was frequently required on both formulations. QHPs with exclusive PA for emtricitabine/tenofovir disoproxil fumarate were higher than other regions, whereas exclusive PA for emtricitabine/tenofovir alafenamide remained at 0%. The number of QHPs with no PA requirements for either formulation was lower.

### Coverage and PA for EHE vs Non-EHE Priority Jurisdiction

For EHE vs non–EHE priority jurisdictions, Table 4 demonstrates coverage and PA requirements of both formulations, only emtricitabine/tenofovir disoproxil fumarate, only emtricitabine/tenofovir alafenamide, and neither. eTable 7 in Supplement 1 reports coverage and PA by EHE vs non-EHE. From 2018 to 2020, coverage of both formulations was higher in EHE jurisdictions compared with non-EHE jurisdictions (difference: 2018, 4.3 percentage points; 2019, 2.4 percentage points; 2020, 2.4 percentage points). Exclusive coverage of emtricitabine/tenofovir disoproxil fumarate was lower in EHE jurisdictions compared with non-EHE jurisdictions (difference: 2018, 4.7 percentage points; 2019, 2.2 percentage points; 2020, 1.4 percentage points). Fewer QHPs required PA on both formulations in EHE areas compared with non-EHE jurisdictions (difference: 2018, 12.0 percentage points; 2019, 7.2 percentage points; 2020, 6.6 percentage points). QHPs with exclusive PA for emtricitabine/tenofovir disoproxil fumarate were higher in EHE areas (difference: 2018, 0.9 percentage points; 2019, 3.5 percentage points; 2020, 29.1 percentage points). QHPs with exclusive PA for emtricitabine/tenofovir alafenamide were rare in both EHE and non-EHE areas (range, 0.0%-0.5%). QHPs without a PA requirement on either formulation were initially more frequent but declined in both EHE and non-EHE jurisdictions (differences: 2018, 10.5 percentage points; 2019, 3.8 percentage points; 2020, 22.6 percentage points).

**Table 4.** Access to F/TDF and F/TAF Among Qualified Health Plans by EHE Priority Jurisdiction Status, 2018-2020

Accessibility by year	Qualified health plans, No./Total No. (%)	
	EHE priority jurisdiction	Non-EHE priority jurisdiction
**Coverage**		
Both		
2018	4936/5232 (94.3)	12 878/14 301 (90.0)
2019	3795/4014 (94.5)	11 962/12 993 (92.1)
2020	4855/5483 (88.5)	4855/16 064 (88.5)
Only F/TDF		
2018	185/5232 (3.5)	1166/14 301 (8.2)
2019	219 (5.5)	994/12 993 (7.7)
2020	385 (7.0)	385/16 064 (7.0)
Only F/TAF		
2018	0	117/14 301 (0.8)
2019	0	0
2020	0	0
Neither		
2018	111/5232 (2.1)	140/14 301 (1.0)
2019	0	37/12 993 (0.3)
2020	243/5483 (4.4)	243/16 064 (4.4)
**Prior authorization requirement** ^a^		
Both		
2018	286/5232 (5.8)	2287/ (17.8)
2019	204/4014 (5.4)	1507/12 993 (12.6)
2020	190/5483 (3.9)	1457/16 064 (10.5)
Only F/TDF		
2018	470 (9.5)/5232	1111/14 301 (8.6)
2019	465/4014 (12.3)	1056/12 993 (8.8)
2020	2612/5483 (53.8)	3411/16 064(24.7)
Only F/TAF		
2018	27/5232 (0.5)	0
2019	0	0
2020	5/5483 (0.1)	0
Neither		
2018	4153/5232 (84.1)	9480/14 301 (73.6)
2019	3126/4014 (82.4)	9399/12 993 (78.6)
2020	2048/5483 (42.2)	8964/16 064 (64.8)

Abbreviations: EHE, Ending the HIV Epidemic initiative; F/TAF, emtricitabine/tenofovir alafenamide; F/TDF, emtricitabine/tenofovir disoproxil fumarate.

^a^ Prior authorization assessed for plans that covered both F/TDF and F/TAF.

### Specialty Tiering

eTable 8 and eTable 9 in Supplement 1 demonstrate specialty drug tiering of QHPs covering emtricitabine/tenofovir disoproxil fumarate and emtricitabine/tenofovir alafenamide for the nation, region, EHE vs non–EHE jurisdiction, and state for 2018 to 2020. Nationally, QHPs were more likely to place emtricitabine/tenofovir disoproxil fumarate on a specialty tier from 2018 to 2020 when compared with emtricitabine/tenofovir alafenamide (difference: 2018, 1.8 percentage points; 2019, 3.7 percentage points; 2020, 4.1 percentage points). Notably, QHPs within the Midwest were more likely to place emtricitabine/tenofovir disoproxil fumarate within a specialty tier compared with other regions.

## Discussion

Although both emtricitabine/tenofovir disoproxil fumarate and emtricitabine/tenofovir alafenamide have indications for biomedical HIV prevention,^29^ this cross-sectional study found geographic differences in coverage and PA for these medications. Although QHPs were more likely to cover emtricitabine/tenofovir disoproxil fumarate than emtricitabine/tenofovir alafenamide, they were also more likely to subject emtricitabine/tenofovir disoproxil fumarate to PA or place it on a specialty tier. Given the broader clinical indication of emtricitabine/tenofovir disoproxil fumarate than emtricitabine/tenofovir alafenamide^30^ and the identical list prices of both drugs in 2020,^31^ this difference in PA and tiering is notable.

We would expect PA and tiering to reflect a combination of clinical factors (eg, PA when there is a limited indication for the drug) and economic factors (eg, PA and/or specialty tiering for expensive drugs).^32^ The PA and tiering trends we found, however, are counter to evidence-based utilization management. PA should have been more prevalent for emtricitabine/tenofovir alafenamide because it has a more limited indication and emtricitabine/tenofovir disoproxil fumarate is the clinically appropriate choice for the vast majority of people without bone or kidney damage who use PrEP. Moreover, generic emtricitabine/tenofovir disoproxil fumarate was entering the market from multiple manufacturers in late spring 2021,^25^ making way for an alternative approximately 60 times cheaper than emtricitabine/tenofovir alafenamide, with a similar safety and efficacy profile for most of PrEP users.^33^ There is little clinical justification for a PA requirement for emtricitabine/tenofovir disoproxil fumarate, and some groups have posited that eliminating the barrier created by PA requirements could help to expedite the prescription process, increase PrEP uptake, and improve adherence.^34^ Although plans have used PA to require clinicians to justify prescribing PrEP for individuals who are at high risk for HIV, clinicians and patients have pushed back on the use of PA in this way, claiming it can be stigmatizing and that discussion of HIV risk should be between patients and clinicians.^35^

One factor contributing to variations in PA and tiering trends could be supplemental rebates negotiated by plans or by pharmacy benefits managers (PBMs) from Gilead Sciences for emtricitabine/tenofovir alafenamide, a common practice within the coverage landscape.^36^ Manufacturer rebate agreements are highly confidential and the specific rebate agreements by medication are not publicly available. However, providing rebates in exchange for formulary placement and the absence of utilization management is a common negotiation practice between PBMs and manufacturers.^37^ In Medicare Part D, where pricing information is more available, researchers analyzed data for emtricitabine/tenofovir disoproxil fumarate and emtricitabine/tenofovir alafenamide and determined that Gilead Sciences offered Medicare Part D plan discounts for emtricitabine/tenofovir alafenamide leading to lower net prices compared with emtricitabine/tenofovir disoproxil fumarate.^38^ Although we do not have the same public data for the QHP market, it is certainly plausible that Gilead Sciences provided similar rebates for emtricitabine/tenofovir alafenamide to persuade plans and PBMs to drop PA and tiering restrictions as it sought to create a PrEP market for emtricitabine/tenofovir alafenamide, which is still under patent, while the patent expired on its branded Federal Trade Commission product.^39^

The disparities in PrEP coverage in the South and other areas of higher HIV burden align with findings from previous research.^23^ Coverage of both formulations was lowest in the South and was lower in EHE jurisdictions than in non–EHE jurisdictions. A reason for this difference may be due to a less restrictive insurance regulatory environment in these states, with less stringent QHP oversight from state department of insurance regulators.^40^

The geographic variations in PA for emtricitabine/tenofovir disoproxil fumarate compared with emtricitabine/tenofovir alafenamide suggest that this utilization management tool is not being used in a uniform or clinically based way. Ultimately, the incongruences across geographic areas and the 2 PrEP formulations add unneeded complexities to prescribing a vital tool for reducing HIV transmission. To end the HIV epidemic, the US must align its health policies in ways that make HIV care and prevention affordable, sustainable, and accessible. Currently, the involvement of for-profit health insurance companies, for-profit pharmaceutical companies, and other for-profit entities causes barriers to a unified approach to ending the HIV epidemic in the US. The for-profit companies’ financial incentives are not always aligned with the EHE initiative, and their influence introduces commercial determinants of health, which have been described by West and Marteau^41^ as “factors that influence health which stem from the profit motive.”

Although there may be instances in which PA is used on the basis of clinical justification,^42^ there is also evidence to suggest that PA can also be an arbitrary way to limit access to necessary medications leading to harmful delays as providers wade through complex override processes.^43^ When considering the timely use and impact of PrEP, any unnecessary delay could possibly lead to a life-changing HIV diagnosis.

It is also important to note that the coverage and PA issues disproportionately impact different groups. The high rates of exclusive PA for emtricitabine/tenofovir disoproxil fumarate in the South and EHE priority jurisdictions will have disproportionately affected cisgender women and transgender men because it was the only FDA-approved PrEP formulation for them during the study.^44^ Barriers to PrEP uptake in the South disproportionately impact Black people, given that more than one-half of Black people in the US live in the South.^45^ This additional barrier to uptake exacerbates the disparity of HIV acquisition along racial lines; 1 in 27 Black men have a lifetime risk of acquiring HIV, whereas 1 in 171 White men have a lifetime risk of acquiring HIV. The disparities are even larger when comparing lifetime risk of acquiring HIV among Black women (1 in 75) with White women (1 in 874).^46^ This PA policy is an example of structural racism, given that Black people would be disproportionally affected by this barrier to PrEP uptake. State insurance regulators may help alleviate this problem if they increase monitoring and oversight of plan design and ensure that plans meet state and federal nondiscrimination requirements.

The possibility that QHP PA and specialty tiering decisions for PrEP may have been influenced by rebate agreements instead of clinical efficacy and safety should also be the subject of regulator action, especially when a pharmaceutical company’s rebate gaming (in conjunction with a massive clinician and direct consumer campaign) could have been used to undercut the generic market for emtricitabine/tenofovir disoproxil fumarate.^39^ A combination of patent reform, drug pricing controls (especially for medications of public health significance) and insurance regulations could guard against access barriers that are based on profit margins, not individual or public health.^47^

Finally, the persistent coverage and cost barriers for PrEP suggest the need for a federal response to PrEP access that parallels the national infrastructure developed for the care and treatment of HIV.^48^ Coupled with insurance reforms, a national PrEP program that is able to negotiate the prices of both medications and laboratories for uninsured and underinsured individuals and expand the PrEP prescriber network could address the growing PrEP health equity crisis in this country.

### Strengths and Limitations

The strengths of this study include its national scope detailing how QHPs covered HIV PrEP from 2018 to 2020. However, this study has limitations. We could not directly assess the impact of disparities in coverage, PA requirements, and specialty tiering on PrEP accessor use. Additionally, we did not have data on the number of subscribers per plan. We also acknowledge and want to highlight that many individuals who would benefit from PrEP face additional barriers to access and adherence, including structural inequities not addressed by our study.

## Conclusions

The findings of this cross-sectional study outline key differences and regional disparities in PA requirements between PrEP formulations. Most notable is the increase in PA requirements in areas with higher rates of HIV diagnoses, a finding that is in direct opposition to the EHE initiative. With evidence-based HIV prevention strategies and the EHE initiative in mind, policymakers, including state insurance regulators, have a vested interest in the accessibility of PrEP. Identification and removal of these barriers, including PA disparities, will be paramount to providing equal access to communities and individuals who would most benefit from PrEP. As the PrEP landscape is continuing to evolve, analyzing access to PrEP and removing systemic barriers is essential.

## Notes

Supplement 1. **eTable 1.** National QHP Coverage and Prior Authorization of F/TDF and F/TAF, 2018-2020 **eTable 2.** Regional QHP Coverage and Prior Authorization of F/TDF and F/TAF, 2018-2020 **eTable 3.** QHP Overall Coverage of F/TDF and F/TAF by State, 2018-2020 **eTable 4.** Overall Prior Authorization of QHPs Covering Both F/TDF and F/TAF by State, 2018-2020 **eTable 5.** QHP Coverage of F/TDF, F/TAF, Both, or Neither, by State, 2018-2020 **eTable 6.** QHP Prior Authorization of F/TDF, F/TAF, Both, or Neither, by State, 2018-2020 **eTable 7.** QHP Coverage and Prior Authorization of F/TDF and F/TAF by EHE Priority Jurisdiction Status, 2018-2020 **eTable 8.** Drug Tiering of QHPs Covering F/TDF and F/TAF for Nation, Regions, and EHE Jurisdiction Status, 2018-2020 **eTable 9.** Drug Tiering of QHPs Covering F/TDF and F/TAF by State, 2018-2020

Supplement 2.
Data Sharing Statement
