# Supplementary material for: Geographic Variation in Qualified Health Plan Coverage and Prior Authorization Requirements for HIV Preexposure Prophylaxis
Source: JAMA Netw Open. 2023 Nov 10;6(11):e2342781. doi: 10.1001/jamanetworkopen.2023.42781 (PMC10638648; doi:10.1001/jamanetworkopen.2023.42781)
Supplement: Supplement 2. — Data Sharing Statement [file jamanetwopen-e2342781-s002.pdf]

Data Sharing Statement

McManus. Geographic Variation in Qualified Health Plan Coverage and Prior Authorization Requirements for HIV Preexposure Prophylaxis. *JAMA Netw Open*. Published November 10, 2023. doi:10.1001/jamanetworkopen.2023.42781

Data
Data available: No

Additional Information
Explanation for why data not available: The data that support the findings of this study include publicly-available data from Robert Wood Johnson's HIX Compare and data received from Ideon under a data use agreement. The data use agreement precludes our sharing of the data. Interested parties are referred to these two avenues to access the data.
